# Increased Autoimmune Diabetes in *pIgR*-Deficient NOD Mice Is Due to a "Hitchhiking" Interval that Refines the Genetic Effect of *Idd5*.*4*

**DOI:** 10.1371/journal.pone.0121979

**Published:** 2015-04-02

**Authors:** Kim R. Simpfendorfer, Richard A. Strugnell, Thomas C. Brodnicki, Odilia L. C. Wijburg

**Affiliations:** 1 The Department of Microbiology and Immunology, The University of Melbourne at The Peter Doherty Institute for Infection and Immunity, Parkville, Victoria, Australia; 2 The Australian Bacterial Pathogenesis Program, The University of Melbourne, Parkville, Victoria, Australia; 3 Immunology & Diabetes Unit, St Vincent’s Institute of Medical Research, Fitzroy, Victoria, Australia; University of Cincinnati College of Medicine, UNITED STATES

## Abstract

Selective breeding to introduce a gene mutation from one mouse strain onto the genetic background of another strain invariably produces “hitchhiking” (i.e. flanking) genomic intervals, which may independently affect a disease trait of interest. To investigate a role for the polymeric Ig receptor in autoimmune diabetes, a congenic nonobese diabetic (NOD) mouse strain was generated that harbors a *Pigr* null allele derived from C57BL/6 (B6) mice. These pIgR-deficient NOD mice exhibited increased serum IgA along with an increased diabetes incidence. However, the *Pigr* null allele was encompassed by a relatively large “hitchhiking” genomic interval that was derived from B6 mice and overlaps *Idd5*.*4*, a susceptibility locus for autoimmune diabetes. Additional congenic NOD mouse strains, harboring smaller B6-derived intervals, confirmed *Idd5*.*4* independently of the other three known susceptibility loci on chromosome 1, and further localized *Idd5*.*4* to an interval proximal to *Pigr*. Moreover, these congenic NOD mice showed that B6 mice harbor a more diabetogenic allele than NOD mice for this locus. The smallest B6-derived interval encompassing the *Pigr* null allele may, however, confer a small degree of protection against diabetes, but this protection appears to be dependent on the absence of the diabetogenic B6 allele for *Idd5*.*4*. This study provides another example of the potential hidden effects of “hitchhiking" genomic intervals and how such intervals can be used to localize disease susceptibility loci.

## Introduction

As a model of human type 1 diabetes, the nonobese diabetic (NOD) mouse strain has proven valuable for characterizing how genes and allelic variation contribute to the pathogenesis of autoimmune diabetes [1]. Genetic outcross studies using NOD mice have identified more than thirty insulin-dependent diabetes (*Idd*) loci that affect the development of autoimmune diabetes [2]. Moreover, selective breeding has been used to generate congenic NOD mouse strains in which specific genomic intervals from non diabetes-prone mouse strains are introduced onto the NOD genetic background to confirm and localize individual *Idd* loci, as well as identify the underlying genes [2,3]. In these congenic studies, the effects upon diabetes onset are due to “naturally” occurring alleles within these laboratory strains of the *Mus* species. Notably, non diabetes-prone mouse strains can harbor alleles that are more diabetogenic than the NOD allele when placed onto the NOD genetic background [4–6].

A complementary strategy to identifying naturally occurring alleles is to introduce engineered null alleles into NOD mice to determine whether a particular gene is critical for the development of autoimmune diabetes. While NOD embryonic stem cells lines are available for gene targeting, relatively few studies have been reported [7–9]. Instead, the conventional method has been to introduce a null allele, which was generated in a different genetic background, into the NOD mouse through a series of selective backcross matings. This method, however, typically introduces a congenic interval of some size that encompasses the null allele from the donor strain. Thus it must be determined if an observed diabetes effect is due to the null allele or the “hitchhiking” congenic interval [10–12].

Susceptibility to type 1 diabetes (T1D) in humans has been shown to coincide with disturbances of the gastrointestinal tract, including increased gastrointestinal permeability, decreased IgA levels and increased inflammation [13,14]. The polymeric Ig receptor (pIgR) actively transports and secretes dimeric IgA and pentameric IgM via intracellular transcytosis to the mucosal lumen [15]. Studies utilizing mice lacking the pIgR have shown that transport of IgM and IgA secretory antibodies (SAbs) is important for protecting the mucosal barrier against pathogens and maintaining tolerance to gastrointestinal commensal flora [15–20]. Given the proposed link between perturbations of mucosal surfaces, commensal flora and the development of T1D [13,14,21,22], we sought to determine the role of pIgR to the development of autoimmune diabetes in the non obese diabetic (NOD) mouse model.

To begin investigating the effect of pIgR upon diabetes pathogenesis, we introduced a *Pigr* null allele generated in C57BL/6 (B6) mice onto the NOD genetic background. *Pigr* is located on chromosome 1, which is known to harbor at least four *Idd* loci: *Idd5*.*1*, *Idd5*.*2*, *Idd5*.*3*, *and Idd5*.*4* [23–26]. We thus generated different congenic mouse strains with or without the *Pigr* null allele to account for the effect of potential contaminating intervals that might overlap an *Idd* locus. Our subsequent study unexpectedly confirmed and localized *Idd5*.*4*, as well as possibly revealing another locus on chromosome 1, as a result of generating *pIgR*-deficient NOD mice with different B6-derived “hitchhiking” intervals.

## Material and Methods

### Mice and ethics statement

NOD/Lt (NOD) and C57BL/6.*Pigr* ^-/-^ (B6.*Pigr* ^-/-^) mouse strains were obtained from the Biological Research Facility in the Department of Microbiology and Immunology at The University of Melbourne. To generate the congenic NOD strains in this study NOD x B6.*Pigr* ^-/-^ F_1_ progeny were backcrossed to NOD mice to generate backcross one generation. Ten subsequent backcrosses were then performed using *Pigr* ^+/-^ backcross progeny and NOD mice. At the 10^th^ backcross generation, mice that were heterozygous for the *Pigr* null allele were intercrossed to generate a NOD mouse strain that was homozygous for the *Pigr* null allele and also carried a B6-derived congenic interval (termed NOD.B6-Chr1^*D1Mit48-D1Mit348*^). To generate additional congenic NOD mouse strains, heterozygous NOD.B6-Chr1^*D1Mit48-D1Mit348*^ mice were intercrossed to generate F_2_ progeny that were screened for recombination events using DNA isolated from tail biopsies and genetic markers that are polymorphic between NOD and B6 mice within the congenic interval for NOD.B6-Chr1^*D1Mit48-D1Mit348*^. Mice were bred and housed under conventional conditions with free access to gamma-irradiated mouse food and sterilized tap water. All animal experiments were approved by The University of Melbourne Animal Ethics and Experimentation Committee (AEC 0703883), and complied with the Prevention of Cruelty to Animals Act (1986) and the National Health and Medical Research Council (NHMRC) Australian Code of Practice for the Care and Use of Animals for Scientific Purposes (1997).

### Genotyping of Mice

DNA samples were extracted from tail biopsies by standard methods and genotyped with polymorphic markers on chromosome 1. Oligonucleotide sequences for polymorphic markers were obtained from Mouse Genome Informatics (www.informatics.jax.org), except for *D1Svi1* (forward oligonucleotide: GGTGGGGCTTGTGTATTGTA, reverse oligonucleotide: TGCATTACTCTGCCCTTTCA). An additional genome-wide screen was performed using DNA from NOD.B6-Chr1^D1Mit48-D1Mit348^ mice and the Autoflex Mass Spectrometer iPLEX GOLD on the Sequenom MassArray by the Australian Genome Research Facility. Data was analyzed using the GeneChip Targeted Genotyping System software. The NOD.B6-Chr1^D1Mit48-D1Mit348^ strain was of the NOD genotype across the whole genome except for those markers within the defined interval on chromosome 1. All subsequent congenic mouse strains described in this study were generated from the NOD.B6-Chr1^D1Mit48-D1Mit348^ strain.

### Detection of IgA

IgA concentration in fecal extracts and serum from mice was measured by ELISA as previously described [27]. Statistical significance of ELISA values between groups was determined using the Mann Whitney *U*-test.

### Diabetes monitoring

Mice were tested once a week for elevated urinary glucose using Diastix reagent strips (Bayer diagnostics). Mice with a positive glycosuria reading (>110 mmol/L) and confirmed by a positive blood glucose reading (>13.0 mmol/L), using Advantage II Glucose Strips (Roche), were diagnosed as diabetic. Pairwise comparisons of diabetes incidence curves were performed using the log-rank test. When more than two comparisons were made between diabetes incidence curves, the P values were adjusted using the Holm method for multiple testing [28].

## Results and Discussion

**Fig 1:**
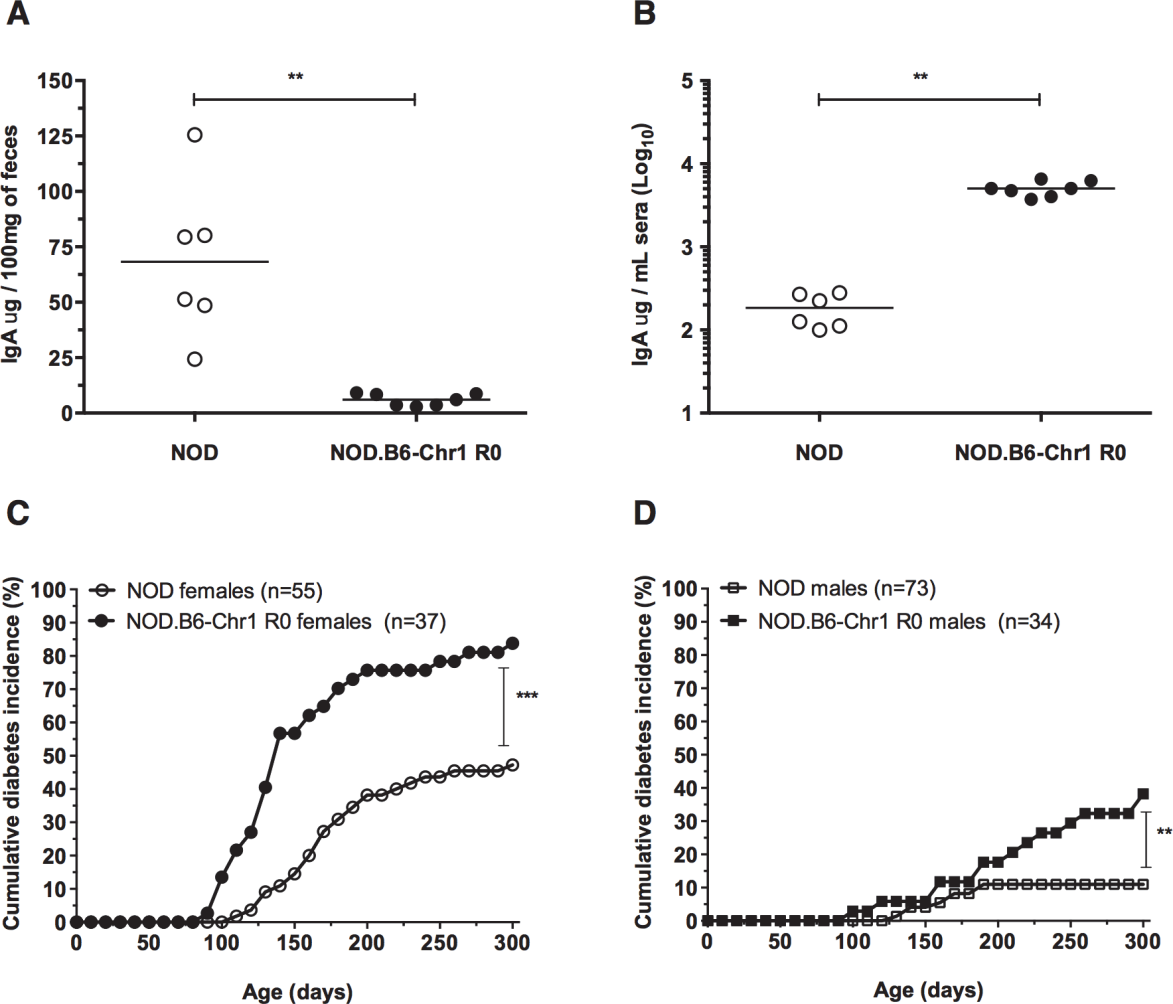
pIgR-deficient NOD mice exhibit altered IgA levels and increased diabetes incidence. IgA concentration in fecal extracts (A) and serum (B) from female NOD and pIgR-deficient NOD mice are shown, mean values are represented by horizontal bars, and statistical significance is represented by ** P = 0.001. The cumulative incidence of diabetes was determined for age-matched female (C) and male (D) cohorts. The statistical significance of pairwise comparisons of diabetes incidence curves are (C) *** P = 7.6x10^-6^, (D) ** P = 0.001.

To investigate the role of pIgR in the development of autoimmune diabetes, we generated a congenic mouse strain that contained the *Pigr* null allele, derived from B6.*Pigr* ^-/-^ mice [27], on the NOD genetic background via serial backcrossing for ten generations. Disruption of *Pigr* on the NOD genetic background resulted in a significant reduction in IgA levels in fecal extracts (as a surrogate measure of IgA in mucosal secretions) compared with age-matched NOD mice that do not harbor the B6-derived *Pigr* null allele (Fig 1A). Conversely, there was a significant increase in IgA levels in serum of pIgR-deficient NOD mice (Fig 1B). These results indicate that disruption of *Pigr* on the NOD genetic background has a similar effect upon IgA secretion as observed in B6.*Pigr* ^-/-^ mice [27]. Furthermore, both female and male pIgR-deficient NOD mice demonstrated increased diabetes incidences compared to age and gender-matched NOD mice, suggesting pIgR plays a role in the development of autoimmune diabetes (Fig 1C and 1D).

During our generation of the pIgR-deficient NOD mouse strain, two new diabetes susceptibility loci (*Idd5*.*3*, *Idd5*.*4*) on chromosome 1 were reported by Wicker and colleagues [25], which brought the total number of *Idd* loci on this chromosome to four, including *Idd5*.*1* and *Idd5*.*2* [23–26]. The defined interval and effect for *Idd5*.*4*, however, was deduced based on interaction with the three other *Idd5* sub-loci [25], but its effect has not yet been confirmed independently of these other loci by a separate congenic NOD mouse strain. Notably, *Pigr* is located within the ~78 Mb interval on chromosome 1 that defines *Idd5*.*4* and for which C57BL/10 (B10) mice were predicted to harbor an allele that increases diabetes susceptibility when placed on the NOD genetic background [25]. As B10 and B6 mouse strains are closely related [29], it was possible that the increased diabetes incidence observed for our pIgR-deficient NOD mice was not caused by pIgR deficiency, but was due to a diabetogenic B6 allele for *Idd5*.*4* within the “hitchhiking” interval encompassing the *Pigr* null allele.

**Fig 2:**
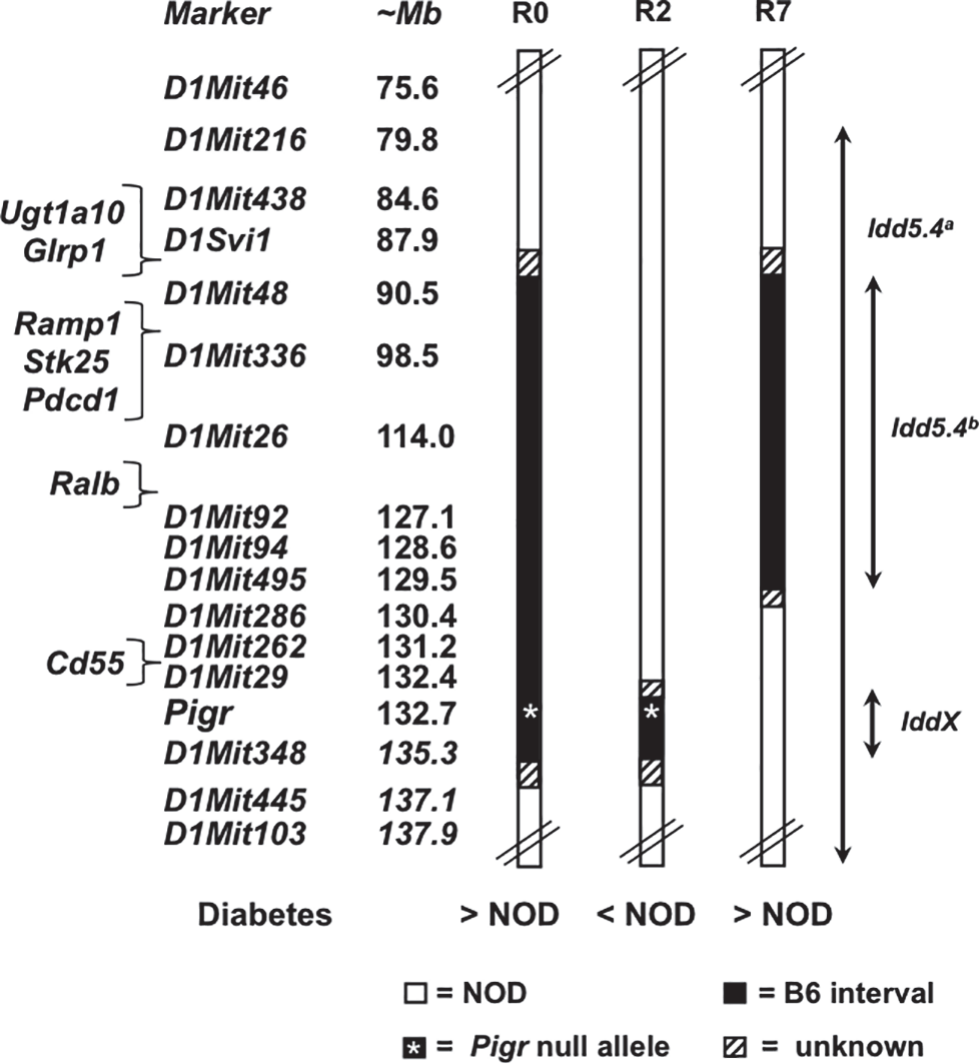
Schematic diagram of mouse chromosome 1 and congenic intervals. Congenic strains names are abbreviated: R0 = NOD.B6-Chr1^D1Mit48-D1Mit348^Pigr^-/-^, R2 = NOD.B6-Chr1^Pigr-D1Mit348^Pigr^-/-^, R7 = NOD.B6-Chr1^*D1Mit48-D1Mit495*^. Diabetes incidence for congenic strains is described relative to NOD mice (>NOD or < NOD, based on Figs 1 and 3). *Idd5*.*4^a^* represents the B10-derived interval defined by Hunter *et al*. [25]; *Idd5*.*4^b^* represents the B6-derived interval, defined by the R7 congenic strain, that confers increased susceptibility to diabetes; *IddX* represents the B6-derived interval harboring the *Pigr* null allele, defined by the R2 congenic strain, that confers protection against diabetes. Marker and gene positions are based on NCBI Bld37, mm9.

It is well known that flanking genomic intervals will accompany a gene mutation from a donor mouse strain (i.e. B6 in this study) when backcrossed onto the NOD genetic background as a result of linkage disequilibrium and recombination hotspots [10–12]. Donor-derived alleles within these so-called “hitchhiking” intervals may affect diabetes incidence independently of the introduced gene mutation. Nevertheless, only one such example for NOD mice has been published to date as far as we are aware. Kanagawa *et al*. showed that reduced diabetes incidence previously reported in NOD mice with a targeted mutation in the IFNγ receptor alpha chain was not due to the lack of the IFNγ receptor. Instead, the reduced diabetes incidence was due to another gene within the 129-derived "hitchhiking" interval that had been introduced along with the mutant *Ifngr1* gene [30]. To determine the size of the “hitchhiking” B6-derived interval in our NOD.B6-*Pigr* ^-/-^ mice, genetic markers that were polymorphic between B6 and NOD mice on chromosome 1 were genotyped (Fig 2). In addition to the *Pigr* null allele (~132.7 Mb), pIgR-deficient NOD mice harbored a B6-derived congenic interval between and including *D1Mit48* (~90.5 Mb) and *D1Mit348* (~134.3 Mb); this congenic strain was subsequently designated as NOD.B6-Chr1^*D1Mit48-D1Mit348*^ *Pigr* ^-/-^ (henceforth abbreviated as NOD.B6-Chr1 R0). This B6-derived congenic interval also overlapped a large portion of the previously defined interval for *Idd5*.*4*, but not the intervals defined for the other three *Idd5* sub-loci on chromosome 1 (Fig 2, [25,26]).

**Fig 3:**
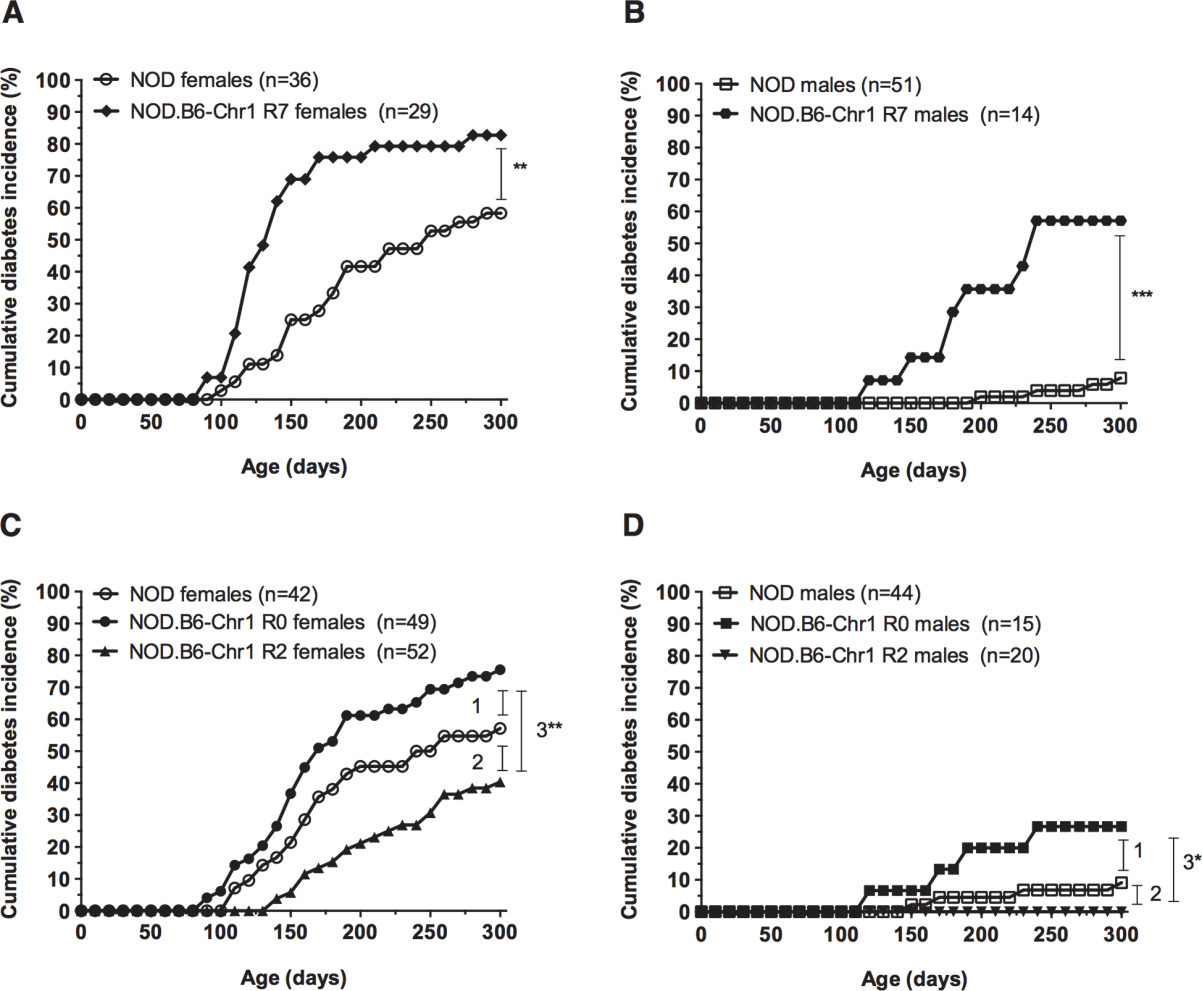
Congenic NOD mouse strains exhibit different diabetes incidences and localize *Idd5*.*4*. The cumulative incidence of diabetes was determined for age-matched cohorts for NOD and NOD.B6-Chr1 R7 females (A) and males (B); and age-matched cohorts for NOD, NOD.B6-Chr1 R0 and NOD.B6-Chr1 R2 females (C) and males (D). Congenic NOD mouse strains were homozygous for their respective B6-derived intervals. Pairwise comparisons of diabetes incidence curves were performed using the log-rank test: (A) ** P = 0.001; (B) *** P = 4.7x10^-6^. For panel (C) and (D), the P values were corrected for multiple testing (i.e. three comparisons): (C) 1: Holm-adjusted P = 0.09, 2: Holm-adjusted P = 0.09, 3**: P = 0.0002; (D) 1: Holm-adjusted P = 0.15, 2: Holm-adjusted P > 0.2, 3*: Holm adjusted P = 0.04.

To dissect the effect of the “hitchhiking” interval encompassing the *Pigr* null allele on diabetes incidence, new congenic mouse strains were derived from the NOD.B6-Chr1 R0 strain and monitored for diabetes onset (Fig 2). Two F_2_ progeny were selected to establish congenic strains that have smaller congenic intervals (Fig 2, NOD.B6-Chr1^*Pigr-D1Mit348*^ *Pigr* ^-/-^ abbreviated as NOD.B6-Chr1 R2, NOD.B6-Chr1^*D1Mit48-D1Mit495*^ abbreviated as NOD.B6-Chr1 R7). These two mouse strains were subsequently monitored for diabetes onset compared to NOD mice. NOD.B6-Chr1 R7 mice exhibited an increase in diabetes incidence compared to NOD mice (Fig 3A and 3B), which was similar to that observed for NOD.B6-Chr1 R0 mice (Fig 1C and 1D). By contrast, neither NOD.B6-Chr1 R2 females nor males exhibited an increased diabetes incidence (Fig 3C and 3D). These results indicate that the *Pigr* null allele is not responsible for the increased diabetes incidence initially observed for NOD.B6-Chr1 R0 mice (Fig 1C and 1D). Instead, the B6-derived R7 interval for Chr1 increased diabetes susceptibility, providing a new "hitchhiker" example for the NOD mouse model that complements the previous example in which a 129-derived "hitchhiking" interval on Chr10 decreased diabetes susceptibility [30]. These B6-derived congenic intervals also indicate that *Idd5*.*4* is located between *D1Svi1* and *D1Mit286*, an ~43Mb genomic interval that is proximal to *Pigr* (Fig 2). Lastly, these results confirm the previous prediction [25], that an allele for *Idd5*.*4* from a non-diabetes prone mouse strain may have a diabetogenic effect independent of the other *Idd5* sub-loci.

This newly defined interval for *Idd5*.*4* is still relatively large and includes a number of candidate genes. For example, *Pdcd1* is located within the defined *Idd5*.*4* interval and encodes the programmed cell death 1 protein (PD-1, Fig 2), which is important for regulating self-reactive T cells and preventing autoimmunity [31,32]. Intriguingly, backcrossing a *Pdcd1* null allele onto the NOD genetic background exacerbated diabetes onset [33]. These PD-1-deficient NOD mice had an early diabetes onset and increased cumulative diabetes incidence (100% by 100 days of age), but the hitchhiking B6-derived interval for this congenic NOD.B6-*Pdcd1* ^-/-^ strain was not defined [33]. Thus, it is possible that the B6-derived interval encompassing the *Pdcd1* null allele is contributing to the observed effect. More recently, Irie *et al*. combined the use of congenic mice with microarrays to analyze expression of genes within their defined *Idd5*.*4* interval [34]. Notably, five genes (*Ugtla10*, *Glrp1*, *Ramp1*, *Stk25*, *Ralb*) localize within our newly defined *Idd5*.*4* interval (Fig 2) and were differentially expressed between activated CD4^+^ T cells from NOD and congenic mice. *Cd55* (*Daf1)* was also identified as a promising candidate gene in their study using B10-derived congenic intervals for *Idd5* sub-loci [34]. In contrast, our B6-derived congenic intervals appear to eliminate *Cd55* from consideration because it does not localize within the congenic intervals for NOD.B6-Chr1 R2 or NOD.B6-Chr1 R7 (Fig 2). It is, however, possible that B10 and B6 mice harbor different sequence for this or other genes within the larger B10-defined *Idd5*.*4* interval, which alters diabetes incidence when placed on the NOD genetic background. New congenic mouse strains with smaller congenic intervals, combined with a haplotype analysis approach [12], will be needed to further localize and refine the list of candidate genes for *Idd5*.*4*, as well as determine if B6 and B10 harbor the same diabetogenic allele for this susceptibility locus.

The effect of pIgR deficiency upon the development of autoimmune diabetes also remains unresolved. Our discovery that introducing a *Pigr* null allele onto the NOD genetic background did not increase diabetes incidence was unexpected because previous reports linked perturbations of mucosal surfaces and commensal flora with the development of T1D [13,14,21,22]. Upon first analysis, the *Pigr* null allele appears to actually confer some degree of protection against diabetes because NOD.B6-Chr1 R2 females have a noticeably lower diabetes incidence curve compared to NOD females (Fig 3C, unadjusted P = 0.05 for single pairwise comparison), and no NOD.B6-Chr1 R2 males became diabetic (Fig 3D). On one hand, it is not clear if this potential protective effect is due to the *Pigr* null allele or due to an independent B6-derived protective allele representing a new *Idd* locus within the ~4 Mb “hitchhiking” congenic interval (Fig 2, noted as *IddX* for the purposes of this study). The *Pigr* null allele, however, still provides a compelling explanation. Deficient production of pIgR has been shown to disrupt mucosal barrier integrity in mice [15,19,27], which may alter the gut microbiota and provide protection against diabetes onset in NOD.B6-Chr1 R2 mice, similar to the effect observed for sex hormones upon gut microbiota that is associated with lower diabetes incidence in male mice [21,35]. On the other hand, a more conservative statistical analysis, which corrects for multiple testing, indicates the difference between NOD and NOD.B6-Chr1 R2 females is suggestive rather than significant (Holm-adjusted P = 0.09). Additional studies are needed to confirm and investigate this potential protective effect, including larger cohorts and the generation of NOD mice harboring the *Pigr* null allele without a “hitchhiking” B6-derived interval (e.g. disruption of *Pigr* using zinc-finger nucleases or CRISPR technology [36,37]).

Our congenic mouse strains also raise the possibility of a genetic interaction between these two loci (*Idd5*.*4* and *Pigr/IddX*) on chromosome 1. It was previously shown by Wicker and colleagues that the diabetogenic B10 allele for *Idd5*.*4* masked the protective effect of the B10 alleles for *Idd5*.*2* and *Idd5*.*3* in congenic NOD mice [25]. Intriguingly, the smaller B6-derived interval harboring the *Pigr* null allele in NOD.B6-Chr1 R2 mice appears to confer a small degree of protection against diabetes (Fig 3C and 3D), but only in the absence of the diabetogenic B6 allele for *Idd5*.*4* (Fig 2). While correction for multiple testing indicates that the difference between NOD and NOD.B6-Chr1 R2 females is only suggestive (Holm-adjusted P value = 0.09), the difference in diabetes incidence between NOD.B6-Chr1 R0 and R2 mice was significant for both sexes (Fig 3C: Holm-adjusted P = 0.0002 for females, Fig 3D: Holm-adjusted P = 0.04 for males). This observation tentatively suggests that the diabetogenic B6 allele for *Idd5*.*4* masks the potential protective effect conferred by the *Pigr/IddX* congenic interval (Fig 2), which corresponds with the diabetogenic B10 allele for *Idd5*.*4* and its ability to mask the protective effect of alleles at other *Idd* loci [25].

It might also be noted that NOD females in our experiments achieved a relatively low cumulative diabetes incidence compared with higher incidences (>80%) reported by other studies [38]. This likely reflects that diabetes monitoring was performed under conventional housing conditions. We postulate that unknown environmental factors (e.g. particular pathogens) may also affect the penetrance of *Idd5*.*4* and/or the *Pigr*/*IddX* loci upon diabetes susceptibility. This appears to be the case given the relatively low diabetes incidence in NOD mice and somewhat different diabetes incidence curve profiles between the two experiments for NOD.B6-Chr1 R0 mice (e.g. Figs 1C and 3C). Further studies in “cleaner” animal rooms (e.g. specific pathogen free or germ-free isolators) are needed to elucidate the contribution of such environmental factors to the penetrance of these diabetes susceptibility loci and pathogenesis in these congenic mouse strains. In either case, it is still clear that the B6-derived interval between *D1Svi1* and *D1Mit286* (Fig 2) increases the risk of diabetes when introduced into the NOD genetic background (Fig 3A and 3B).

In summary, this study took advantage of new congenic mouse strains to further evaluate loci on chromosome 1 that affect diabetes incidence in NOD mice. Conventionally, gene mutations have been backcrossed onto the NOD genetic background because efficient NOD embryonic stem cell lines were not available for targeted gene disruption. The disadvantage of this approach is that the resulting mutant NOD mouse strains harbor a “hitchhiking” congenic interval that may alter disease pathogenesis independent of the introduced gene mutation [10–12]. We show here the power of congenic mouse strains to determine the effect of a “hitchhiking” interval. By dissecting the original *Pigr* “hitchhiking” interval with new congenic mouse strains, we confirmed for the first time that *Idd5*.*4* has an independent effect upon diabetes susceptibility in NOD mice, whereas previous work by others showed its effect only in combination with other *Idd5* sub-loci [25]. Moreover, we were able to further localize *Idd5*.*4* on Chr1 and demonstrate that a non-diabetogenic mouse strain (i.e. B6) harbors an allele for *Idd5*.*4* that is more diabetogenic than NOD mice. These findings add to the accumulating evidence that different combinations of alleles, outside the NOD subset, can affect the risk for developing diabetes (e.g. [6,25,26,39–41]). Our study also highlights once again the potential hidden effects of “hitchhiking” genomic intervals that must be taken into account when interpreting the effects of gene mutations that have been backcrossed onto a different genetic background [10–12]. Such effects, however, can be used to refine the location and genetic contribution of previously described diabetes susceptibility loci.
